# Association of *CDKN2A/B* and *MTAP* deletions in adult-type diffuse gliomas

**DOI:** 10.1093/jnen/nlag007

**Published:** 2026-03-09

**Authors:** Blake A Ebner, Cristiane M Ida, Thomas M Kollmeyer, Robert B Jenkins, Cinthya J Zepeda Mendoza, Caterina Giannini, Jorge A Trejo-Lopez, Aditya Raghunathan

**Affiliations:** Department of Laboratory Medicine and Pathology, Mayo Clinic, Rochester, Minnesota USA; Department of Laboratory Medicine and Pathology, Mayo Clinic, Rochester, Minnesota USA; Department of Laboratory Medicine and Pathology, Mayo Clinic, Rochester, Minnesota USA; Department of Laboratory Medicine and Pathology, Mayo Clinic, Rochester, Minnesota USA; Department of Laboratory Medicine and Pathology, Mayo Clinic, Rochester, Minnesota USA; Department of Laboratory Medicine and Pathology, Mayo Clinic, Rochester, Minnesota USA; Department of Laboratory Medicine and Pathology, Mayo Clinic, Rochester, Minnesota USA

**Keywords:** adult-type diffuse gliomas, astrocytoma, *CDKN2A/B;* glioblastoma, homozygous deletion, *MTAP;* oligodendroglioma

## Abstract

In adult-type diffuse gliomas *CDKN2A* and/or *CDKN2B* (*CDKN2A/B*) deletions often co-occur with deletion of *MTAP*, suggesting that MTAP immunohistochemistry (IHC) may be a surrogate marker of *CDKN2A*/*B* status. However, the association between *CDKN2A/B* and *MTAP* deletion at the genomic level remains unknown. We assessed *CDKN2A/B* and *MTAP* deletions by chromosomal microarray in 333 adult-type diffuse gliomas and performed MTAP IHC on a subset (n = 63). *CDKN2A/B* and *MTAP* deletions were detected in 216 and 215 cases, respectively, and were concurrent in 99.5% (215/216). While most tumors with *CDKN2A/B* homozygous deletion (n = 148) showed concurrent *MTAP* homozygous deletion (108/148; 73.0%), a subset harbored *MTAP* heterozygous deletion (39/148; 26.4%). By analyzing the size of the chromosomal alterations, we demonstrate that initial large chromosomal 9p losses result in concurrent heterozygous deletion of *CDKN2A/B* and *MTAP* whereas smaller “second hit” deletions leading to homozygous *CDKN2A/B* deletion do not always encompass the *MTAP* locus. Discordant *CDKN2A/B* and *MTAP* tumors affect the association between MTAP IHC and copy number status of *MTAP* and *CDKN2A/B*. These findings suggest that adult-type diffuse gliomas, regardless of *IDH* status, follow a stereotypic pathway involving concurrent *CDKN2A/B* and *MTAP* heterozygous deletion but may diverge for *CDKN2A/B* and *MTAP* homozygous deletion.

## INTRODUCTION

The copy number status of *CDKN2A* and *CDKN2B* is a critical prognostic and diagnostic biomarker in multiple primary central nervous system (CNS) neoplasms; homozygous deletion of *CDKN2A* and/or *CDKN2B* (*CDKN2A/B*) is associated with aggressive clinical behavior and reduced overall survival (1–6). In the latest edition of the CNS WHO tumor classification (5^th^), *CDNK2A/B* homozygous deletion is a molecular feature sufficient to warrant a high-grade designation in IDH-mutant astrocytomas (CNS WHO grade 4) and meningiomas (CNS WHO grade 3) (7). Under the CNS WHO 2021, *CDKN2A/B* homozygous deletion is not required for grading oligodendroglioma, IDH-mutant and 1p/19q-codeleted, but has been associated with shorter survival and when present in a tumor with borderline or ambiguous histology, may warrant a Grade 3 designation. As a diagnostic marker, the presence of *CDKN2A*/*B* homozygous deletion is included in the diagnostic criteria for pleomorphic xanthoastrocytoma and high-grade astrocytoma with piloid features (3, 4, 8, 9) and its absence is listed in the diagnostic criteria for diffuse low-grade glioma, MAPK pathway-altered (7). Thus, assessment of the *CDKN2A/B* copy number status is increasingly necessary in routine neuropathology practice. Testing for *CDN2KA/B* copy number status at present is primarily performed via expensive and labor-intensive techniques including fluorescence in-situ hybridization (FISH) or high-resolution genome-wide analysis such as chromosomal microarray. Assessment of the *CDNK2/B* copy number status by routine immunohistochemistry (IHC) may, therefore, be more accessible to the routine evaluation of CNS and non-CNS neoplasia such as a spectrum of gliomas, meningioma, and mesothelioma.

The *CDKN2A* and *CDKN2B* genes reside in tandem on chromosome 9p21.3 and encode for three tumor suppressor genes that have been implicated in the tumorigenesis in diverse group of malignant neoplasms (10–13). *CDNK2A* encodes for two proteins of distinct sequence and function: cyclin-dependent kinase inhibitor 2A, also known as p16-INK4A, and tumor suppressor ARF, also known as p14ARF. *CDKN2B* encodes for cyclin-dependent kinase 4 inhibitor B, also known as p14-INK4b/CDK4I and p15-INK4b (14–18). p16-INK4A, p15-INK4b, and p14-INK4b/CDK4I each regulate distinct signaling cellular pathways, including p53, pRb, and CDK4/CDK6. Reports evaluating p16 IHC as a surrogate marker for assessing *CDNK2A* status have thus far been variable in terms of specificity, ranging from 71 to 96% (19–22). The gene, S-methyl-5'-thioadenosine phosphorylase, also known as *MTAP*, is situated approximately 100 kilobases distal telomeric to *CDNK2A* on 9p21.3. As a consequence of its proximity to *CDKN2A/B*, the use of MTAP IHC has been previously explored as an alternative surrogate marker for the *CDKN2A*/*B* copy number status in gliomas (21–26).

These reports have established MTAP IHC as a sensitive and specific surrogate marker of *CDKN2A/B* copy number status. However, the genomic relationship between *CDKN2A/B* and *MTAP* deletion remains unclear. In this study, we sought to: a) evaluate the association between the *MTAP* and *CDKN2A/B* loci alterations at the genomic level in adult-type diffuse gliomas utilizing chromosomal microarray analysis and b) correlate MTAP expression by IHC to the various *MTAP* and *CDKN2A/B* copy number alterations involving these loci.

## METHODS

Following approval by the Institutional Review Board and as part of an institutional initiative, consecutive primary CNS tumors had been comprehensively characterized by targeted next-generation sequencing and chromosomal microarray analysis. Chromosomal microarray was performed using the OncoScan CNV Plus Assay (Thermo Fisher Scientific, Waltham, MA) (27). The OncoScan is a multiplexed molecular inversion probe assay that utilizes oligonucleotides probes targeting unique single nucleotide polymorphic base pairs and interrogates copy number alterations in a whole genome array. The functional resolution across the genome is roughly 100 kilobases for non-mosaic deletions and duplications. Thresholds for clinically significant copy number events include deletions larger than 1 megabase, duplications larger than 2 megabases, and copy-neutral loss of heterozygosity (cnLOH) larger than 10 megabases.

To assess copy number alterations of chromosome 9, the chromosomal microarray data were closely annotated for the overall copy number alterations, ploidy, and length of the copy number alterations to approximate deletion sizes. For the genomic analysis, because *CDKN2A/B* homozygous deletion is a distinct grade defining event in IDH‑mutant astrocytoma with strong adverse prognostic impact, we analyzed *CDKN2A/B* homozygous deletion as a separate category. In contrast, tumors with hemizygous deletion and copy‑neutral loss of heterozygosity were grouped together in the analysis, as both represent a monoallelic loss state. With the primary goal of understanding the overall genetic architecture changes whereby a second hit could yield functional biallelic inactivation, we assumed that tumors with hemizygous deletion and cnLOH would be analytically equivalent. All tumors with homozygous and/or heterozygous deletions as well as cnLOH involving the entire or part of the *CDKN2A*, *CDKN2B* and *MTAP* genes were included in the analysis. In tumors with homozygous deletion of *CDNK2A/B*, the cumulative length of the heterozygous, and homozygous deletions was determined by assuming a deletion was “contiguous” if the heterozygous deletions were within 25 kb of the homozygous deletion of *CDKN2A/B*. For the contiguity analysis, a small subset tumors (n = 10) showed complex combinations of gains or losses in the 9p24 through 9p21 region; if gain alterations were present in the continuous chain, they were treated as breaks. For the copy number events analysis, all chromosome 9 alteration annotations (including gain/amplifications, losses, and cnLOH) were counted on each tumor, alterations on other chromosomes were excluded. Heterozygous deletions were visualized using Chromomap v1.4.1 (28).

Mutational status for 118 CNS tumor-related genes, including *CDKN2A* and *CDKN2B*, was evaluated by the custom amplicon-based Mayo Clinic targeted neuro-oncology next-generation sequencing (NGS) panel, as previously described (29).

The genetic analysis of the 333-patient cohort showed that *MTAP* and *CDKN2A/B* deletions are not always concordant. Next, we sought to specifically evaluate how MTAP IHC would perform in such cases. Therefore, we performed MTAP immunostaining on a subset of cases from the 333-patient cohort and we selectively biased the cohort to include a large proportion of tumors with discordant *MTAP* and *CDKN2A/B* alternations. The IHC cohort of 63 tumors was generated as follows: first, we generated a balanced distribution of cases by *CDKN2A/B* status, including cases with intact copy number state (n = 17), heterozygous deletion (n = 21), and homozygous deletion (n = 25) of *CDKN2A/B*. Considering that that hemizygous deletion and cnLOH *CDKN2A/B* may differ in expression dosage, cnLOH tumors were excluded for this analysis. Second, we enriched our IHC cohort with discordant deletions of *MTAP* and *CDKN2A/B*. Our prior analysis demonstrated that the discordant *MTAP* and *CDKN2A/B* tumors exclusively arise in the *CDKN2A/B* homozygous deleted tumors; therefore, within the cases selected for *CDKN2A/B* homozygous deletion we included 11 tumors with concordant *MTAP* and *CDKN2A/B* homozygous deletion and 14 tumors with discordant *MTAP* and *CDKN2A/B* deletions. The discordant tumors included 11 tumors with homozygous *CDKN2A/B* deletion with heterozygous *MTAP* deletions and 3 tumors with homozygous *CDKN2A/B* deletion with partial homozygous deletion of the *MTAP* gene.

Immunohistochemical staining for MTAP (1:400; Abnova, 11475-1-AP) was performed on representative formalin-fixed paraffin-embedded tissue sections utilizing standard techniques. We used a polyclonal MTAP antibody (Proteintech 11475‑1‑AP) to maximize epitope breadth, hypothesizing improved detection of truncated MTAP in partially deleted tumors. The immunostained slides were independently reviewed in a blinded fashion by three board certified neuropathologists (A.R., C.G., and J.T.). External positive controls (colon and kidney) were included for each antibody, as well as primary antibody omission controls. Internal positive controls were assessed in each tumor for the expected immunoreactivity, such as endothelium and reactive astrocytes, and within the adjacent non-neoplastic parenchyma (if present). Cytoplasmic or nuclear expression MTAP within tumor cells was assessed on a three-point scale: 0 for expression less than 40% of tumor cells, “1” for expression present in 40-80% of tumor cells, and “2” for expression in 80-100% of the tumor cells. For each pathologist we quantified the binary/ordinal association between MTAP IHC loss and copy number loss using the φ coefficient, in the presence or absence of the discordant cases. For the binary association analyses we used a strict loss definition, with MTAP IHC score 0 = loss, and MTAP IHC score 1 or 2 = intact. *MTAP* and *CDKN2A/B* copy number was coded as ordinal levels (0 = homozygous deletion, 1 = hemizygous deletion, and 2 = intact). Copy number status also used a strict loss definition with *MTAP* and *CDKN2A/B* copy number 0 = loss, and 1 or 2 = intact. To determine for each pathologist if φ coefficients (including vs excluding discordant cases) were statistically different, a permutation test was performed to determine if removing the discordant tumors produces a larger increase in φ than would be expected than by random chance (5,000 iterations, two‑sided p‑value).

**Figure 1.**
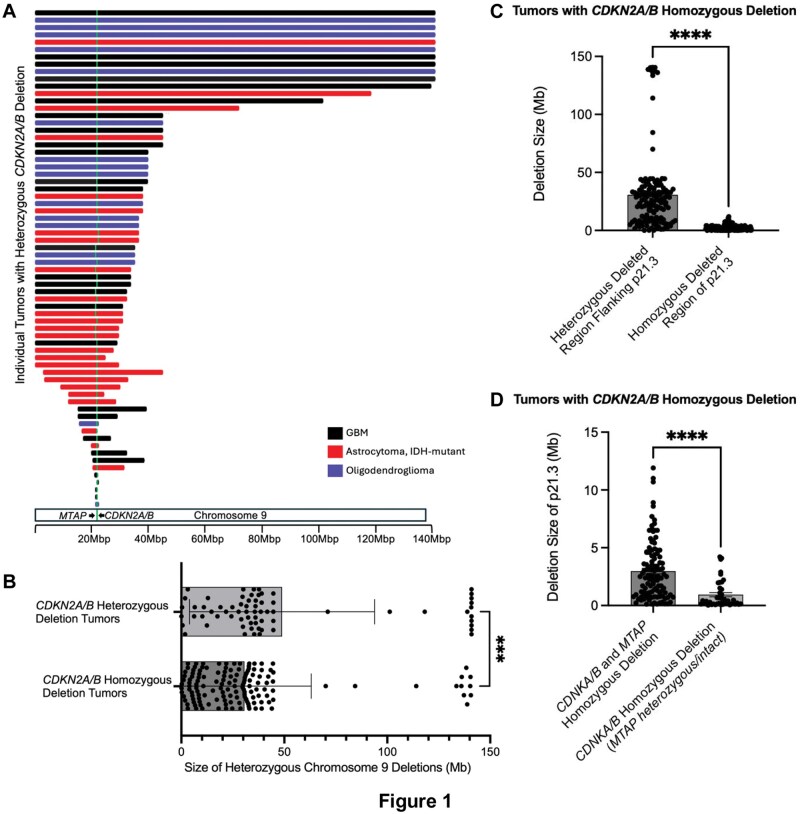
Chromosome 9 deletion sizes in tumors with *CDKN2A/B* heterozygous and homozygous deletions. (A) Chromosome 9 deletions in tumors with *CDKN2A/B* heterozygous deletion or copy-neutral loss of heterozygosity: This figure maps the size and location of the chromosome 9 deletions in the 68 tumors *CDKN2A/B* heterozygous deletion or copy-neutral loss of heterozygosity. Locus of *CDKN2A/B* and *MTAP* is shown by green bar. (B) Graph shows the size of the cumulatively heterozygously deleted regions of chromosome 9 in tumors with heterozygous or homozygous *CDKN2A/B* deletions (mean deletion size 48.9 and 30.6 Mb, respectively). (C) In tumors with *CDKN2A/B* homozygous deletion, the size of the heterozygously deleted regions of chromosome 9 is significantly larger than the homozygous deleted region of *CDKN2A/B* (mean 30.6 and 2.4 Mb, respectively). (D) The size of the *CDKN2A/B* locus homozygous deletion is significantly smaller in tumors with *CDKN2A/B* homozygous deletion without concordant homozygous *MTAP* deletion, relative to tumors with homozygous deletion of both genes (mean 3.0 and 0.94 Mb, respectively). *** p < 0.0002, and **** p <0.0001 determined by Mann-Whitney U Test.

**Figure 2.**
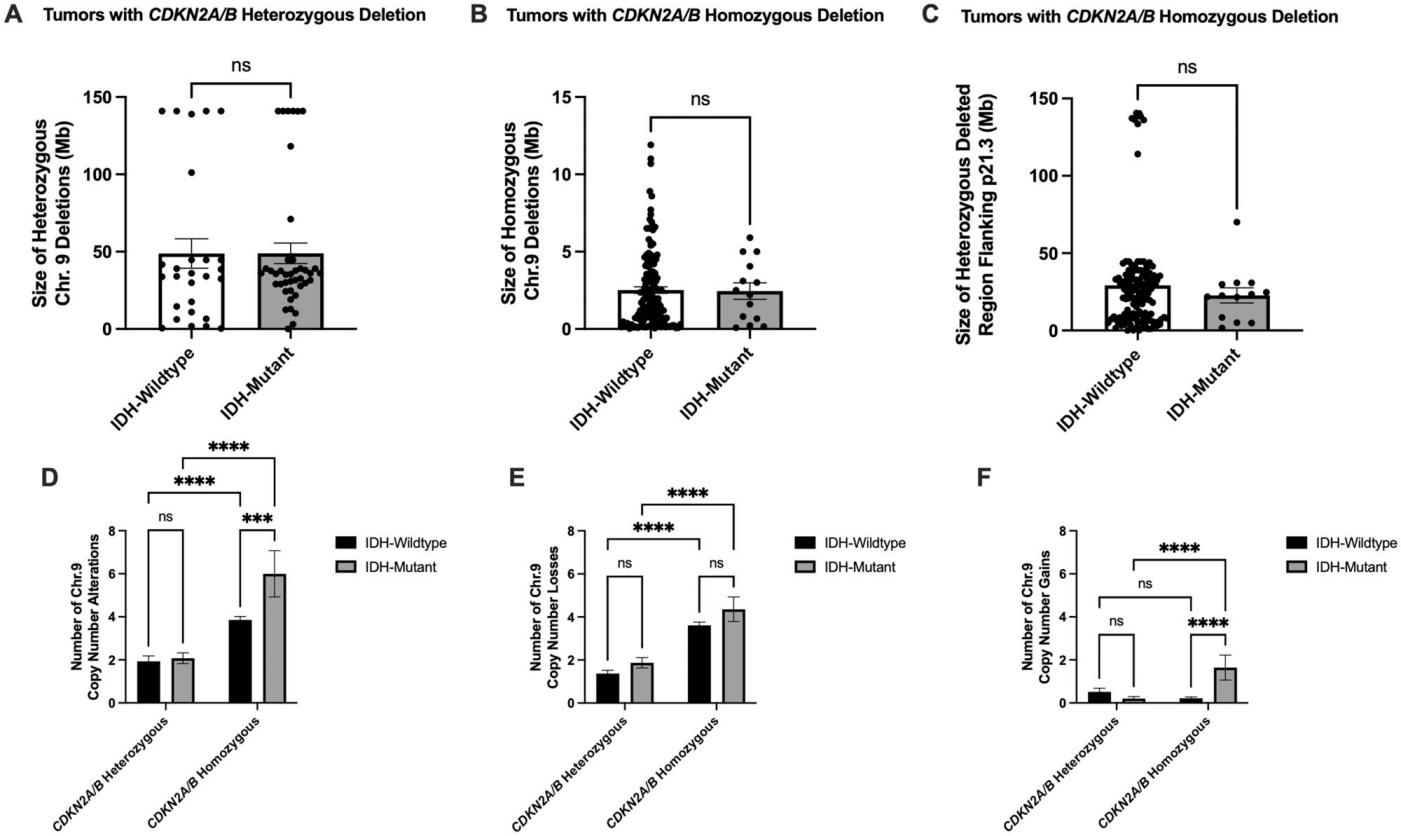
Chromosome 9 alterations in IDH-wildtype and IDH-mutant tumors with *CDKN2A/B* heterozygous and homozygous deletions. (A) Size of the cumulatively heterozygously deleted regions of chromosome 9 in IDH-wildtype and mutant tumors with heterozygous *CDKN2A/B* deletions (mean deletion size 48.9 and 50.0 Mb, respectively). (B, C) In tumors with homozygous *CDKN2A/B* deletion, the size of homozygously deleted regions (B; mean 2.5 Mb in both groups) and heterozygously deleted regions (C; mean 29.1 Mb and 22.7 Mb, respectively) did not differ significantly between IDH subtypes. (D) Total number of chromosome 9 alterations (including losses, cnLOH, gains/amplifications) in IDH-wildtype and IDH-mutant tumors with heterozygous/homozygous *CDKN2A/B* deletions. (E) Both IDH-wildtype and mutant tumors with homozygous *CDKN2A/B* deletions show an increase in chromosome 9 copy number losses relative to tumors with *CDKN2A/B* heterozygous deletion. (F) IDH-mutant tumors with homozygous *CDKN2A/B* deletions demonstrate a slight increase in chromosome 9 gain events. **** p < 0.0001, and *** p = 0.008 determined using two-way ANOVA followed by Tukey’s multiple comparison post-tests (D-F).

Mann-Whitney U Test was used to calculate statical differences between the deletion sizes in the different tumor groups (Figs. 1, 2A-C). Group comparisons were analyzed using two-way ANOVA followed by Tukey’s multiple comparison post-tests (Fig. 2D-F). All analyses were performed utilizing Python 3.12 and GraphPad PRISM (version 10).

## RESULTS

### Cohort characteristics and CDKN2A/B copy number status

The cohort included primary CNS tumors from 333 patients, 332 (99.7%) of whom were adults (median age, 52 years; range 16-93), with 4:7 female to male ratio. The majority of the tumors (301; 90.4%) were the first-time tissue diagnosis with a smaller subset of recurrent tumors. The tumor entities were composed of adult-type diffuse gliomas including glioblastoma, IDH-wildtype (202; 60.6%), astrocytoma, IDH-mutant (82; 24.6%) and oligodendroglioma, IDH-mutant and 1p/19q-codeleted (49; 14.7%) (Table 1). The cohort included tumors corresponding to CNS WHO grade 2 (n = 53; 15.9%), grade 3 (n = 58; 17.4%) and grade 4 (n = 222; 66.7%)

**Table 1.** *CDKN2A/B* and *MTAP* copy number status in in adult-type diffuse gliomas

		*CDKN2A/B* Status			*MTAP* Status		
	N (%)	Homozygous deletion	Heterozygous deletion^1^	Intact	Homozygous deletion	Heterozygous deletion	Intact
Glioblastoma, IDH-wildtype	202 (60.6%)	134 (66.3)	27 (13.4)	41 (20.3)	97 (48.0)	63 (31.2)	42 (20.8)
Astrocytoma, IDH-mutant	82 (24.6%)	12 (14.6)	25 (30.5)	45 (54.9)	10 (12.2)	27 (33.0)	45 (58.5)
*CNS WHO Grade 2, N= 24 (29.3%)*		0	5 (20.8)	19 (79.2)	0	5 (20.9)	19 (79.2)
*CNS WHO Grade 3, N = 38 (47.6%)*		0	19 (50.0)	19 (50.0)	0	19 (50.0)	19 (50.0)
*CNS WHO Grade 4, N = 20 (24.4%)*		12 (60.0)	1 (5.0)	7 (35.0)	10 (50.0)	3 (15.0)	7 (35.0)
Oligodendroglioma, IDH-mutant, 1p/19q-codeleted	49 (14.7)	2 (4.1)	16 (32.7)	31 (63.3)	1 (20.4)	17 (34.7)	31 (63.3)
*CNS WHO Grade 2, N = 29 (59.2%)*		0	7 (24.1)	22 (75.9)	0	7 (58.6)	22 (78.9)
*CNS WHO Grade 3, N = 20 (40.8%)*		2 (10.0)	9 (45.0)	9 (45.0)	1 (5.0)	10 (50.0)	9 (45.0)
Total N:	333	148	68	117	108	107	118

1 Including 8 cases with *CDKN2A/B* copy-neutral loss of heterozygosity.

Among all 333 cases, 216 (65.0%) tumors had *CDKN2A/B* copy number alterations, which included 148 *CDKN2A/B* homozygous deletions, 60 *CDKN2A/B* heterozygous deletions and 8 cnLOH. By NGS, only two tumors (1 glioblastoma, IDH-wildtype and 1 astrocytoma IDH-mutant) were found to have a clinically relevant mutation in *CDKN2A*. The integrated diagnoses and *CDKN2A/B* copy number status are detailed in Table 1. When considering cnLOH functionally equivalent to tumors with heterozygous deletion, tumors with *CDKN2A/B* homozygous and heterozygous deletions (n = 148; n = 68) respectively included glioblastoma, IDH-wildtype (n = 134; n = 27), astrocytoma, IDH-mutant (n = 12; n = 25), and oligodendroglioma, IDH-mutant and 1p/19q-codeleted (n = 2; n = 16). There was not a significant difference in the proportion of tumors with homozygous and heterozygous *CDKN2A/B* deletions between primary and recurrent tumors (68.7% vs. 67.7%, p = 1.0).

*CDKN2A* and *CDKN2B* copy number alterations were evaluated for a correlation between their respective copy number alterations. Overall, 215 (of 216; 99.5%) tumors had concomitant *CDKN2A* and *CDKN2B* heterozygous and/or homozygous deletions or cnLOH. Of 71 tumors with *CDKN2A* heterozygous deletion or cnLOH, 70 (98.6%) showed concurrent *CDKN2B* deletion (3 homozygous and 59 heterozygous) or cnLOH (n = 8). Of 145 cases with *CDKN2A* homozygous deletion, 141 (96.0%) had concurrent *CDKN2B* homozygous deletion, while 4 (2.8%) showed *CDKN2B* heterozygous deletion. Of the 144 cases with *CDKN2B* homozygous deletion, 141 (97.9%) had concurrent *CDKN2A* homozygous deletion, while 3 (2.1%) showed *CDKN2A* heterozygous deletion. These results demonstrate that the *CDKN2A* and *CDKN2B* copy number changes frequently co-occur and are of the same type.

### Correlation between CDKN2A/B and MTAP copy number alterations

The association between *CDKN2A/B* and *MTAP* copy number alterations is shown in Table 2. Among the 216 tumors with *CDKN2A/B* heterozygous and/or homozygous deletions or cnLOH, 215 (99.5%) had concurrent *MTAP* deletions. The single tumor without concurrent *MTAP* deletion, had two focal deletions (0.068Mb and 0.099Mb) resulting in isolated homozygous deletion of *CDNK2B.* Thus, all cases with *CDKN2A* heterozygous and/or homozygous deletions or cnLOH had an *MTAP* deletion. All 215 cases with *MTAP* deletions had concurrent *CDKN2A/B* copy number alterations, and all 117 tumors with intact *CDKN2A/B* copy number status also had intact *MTAP* copy number status.

**Table 2.** Correlation of *CDKN2A/B* and *MTAP* copy number status

	CDKN2A/B Homozygous Deletion N (%)	CDKN2A/B Heterozygous Deletion/ LOH	CDKN2A/B Intact
*MTAP* Homozygous Deletion	108 (73.0%)	0	0
Entire *MTAP* deletion	94		
Partial *MTAP* deletion	14		
*MTAP* Heterozygous Deletion*/LOH*	39 (26.4%)	68 (100%)	0
Entire *MTAP* deletion	25	66	
Partial *MTAP* deletion	14	2	
*MTAP* Intact	1 (0.7%)	0	117 (100%)

Within the 148 tumors harboring *CDKN2A/B* homozygous deletion, 73.0% (n = 108) had concurrent *MTAP* homozygous deletion. Most of these *MTAP* homozygously deleted cases showed homozygous deletion of the entire *MTAP* gene but 14 tumors (13.0%) showed partial *MTAP* homozygous deletion. Concordant *CDKN2A/B* and *MTAP* homozygous deletions were identified in 83.3% of astrocytoma, IDH-mutant (10/12) and 72.4% of glioblastoma, IDH-wildtype (97/134). Of the 10 glioblastoma, IDH-wildtype tumors defined by molecular criteria, 80% had concordant *MTAP* homozygous deletions (8/10). Only 2 oligodendrogliomas harbored *CDKN2A/B* homozygous deletions, of which 1 had a concurrent *MTAP* homozygous deletion (50%).

To evaluate the underlying genomic architecture of copy number alterations associated with concordant *CDKN2A/B* and *MTAP* deletions, we assessed the changes on chromosome 9 among tumors with *CDKN2A/B* and *MTAP* heterozygous deletion (including 27 glioblastomas, 25 astrocytomas, 16 oligodendrogliomas). The majority of the cases (n = 52, 76.4%) demonstrated contiguous large deletions (>20 Mb) of chromosome 9, spanning across 9p21 and including *MTAP* and *CDKN2A/B* (mean 48.9 Mb ± 44.6 [SD]). Interestingly, 41 tumors (60.3%) with heterozygous *CDKN2A/B* deletions harbored a distal telomeric deletion involving 9p24, specifically in the region of chr9:204737 (Fig, 1A). In tumors with homozygous *CDKN2A/B*, the homozygously deleted segments of *CDKN2A/B* were typically small (mean 2.4 Mb) and associated with immediately flanking proximal and distal larger heterozygous deletions (mean cumulatively measuring 30.6 Mb) (Fig. 1C). A comparison of these flanking heterozygous deletions in homozygous and heterozygous deleted *CDKN2A/B* tumors shows that in tumors with *CDKN2A/B* heterozygous deletion, the average size of heterozygous deletion is 48.9 Mb (Fig. 1B). In tumors with *CDKN2A/B* homozygous deletion, a similarly large, albeit it smaller, portion of chromosome 9 flanking *CDKN2A/B* is also heterozygously deleted (30.6 Mb). These findings suggest that the homozygous *CDKN2A/B* deletion arises from a small second hit within a larger pre-existing hemizygous deletion with the remaining heterozygously deleted portion of 9p likely representing the residual heterozygous deletion similar in size to the original, but slightly shorter. The heterozygous deleted *CDKN2A/B* tumors show a striking somewhat bi-modal distribution in the 9p deletion size (ranging from 25-50Mb and 140Mb). Interestingly, the homozygous *CDKN2A/B* tumors show the above, and an additional cluster of smaller heterozygous deletions (<15 Mb). Only 5 of 157 tumors, all glioblastoma, IDH-wildtype, did not show detectable heterozygous deletions flanking the homozygous deleted region of *CDKN2A/B*. As expected, tumors with homozygous deletion of *CDKN2A/B* without concordant homozygous loss of MTAP showed significantly smaller deletions *CDKN2A/B* locus of compared to tumors with homozygous deletion of both genes (mean deletion size of 0.94 and 3.0 Mb, respectively) (Fig. 1D).

Next, we determined if there were any differences between IDH-wildtype (histologically and molecularly defined GBMs) and IDH-mutant tumors (astrocytoma IDH-mutant and oligodendroglioma). We observed no differences in the size of the heterozygous chromosome 9 deletions in the IDH-wildtype vs IDH-mutant tumors with heterozygous deletion of *CDKN2A/B (*Fig. 2A*).* Likewise, in tumors with *CDKN2A/B* homozygous deletion, no difference was observed in IDH-wildtype vs IDH-mutant tumors either in the homozygous deletion size of 9p or the heterozygous deletions flanking the homozygously deleted *CDKN2A/B* (Figs. 2B, C). When we broadly examined the total number of chromosome 9 copy number alterations in IDH-wildtype and mutant tumors, both types demonstrated the expected increase in the number alterations in the tumors harboring a *CDKN2A/B* homozygous deletion (Fig. 2D). In both IDH-wildtype and mutant tumors with *CDKN2A/B* homozygous deletion, the total increase in the number of chromosome 9 alterations, which is primarily due to increases in copy number losses (Fig. 2E). The IDH-mutant tumors with a *CDKN2A/B* homozygous deletions showed a relatively small increase in chromosome 9 copy number gains compared to IDH-mutant tumors (1.6 vs 0.2; Fig. 2F). In all, these findings suggest that the IDH mutation does not significantly alter the size of the *CDKN2A/B* deletion or induce a significant alteration to chromosome 9 stability relative to IDH-wildtype tumors.

### Discordant tumors alter the MTAP IHC association to *MTAP* and *CDKN2A/B* copy number status

**Figure 3.**
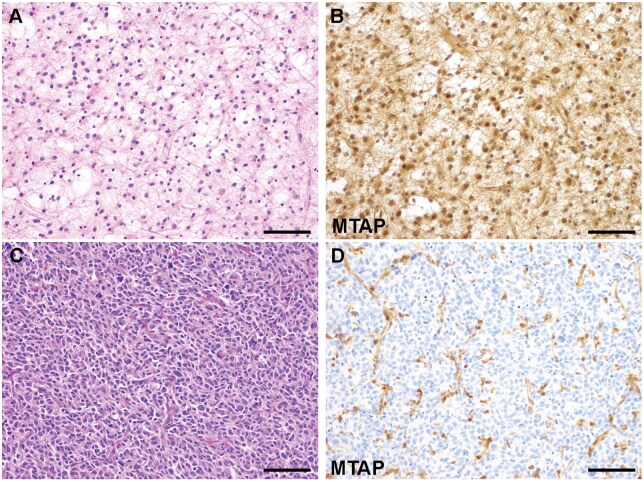
Illustrative examples of MTAP expression in Astrocytoma, IDH-mutant, without and with *CDKN2A/B* homozygous deletion. (A, B) Astrocytoma, IDH-mutant, without *CDKN2A/B* deletion (CNS WHO grade 2) (A, H&E, 200x), and preserved cytoplasmic and nuclear MTAP expression (B, 200x). (C, D) Astrocytoma, IDH-mutant, with *CDKN2A/B* homozygous deletion (CNS WHO grade 4) (C, H&E, 200x), and loss of cytoplasmic and nuclear MTAP expression (D, 200x). Scale bar, 100 μm.

**Figure 4.**
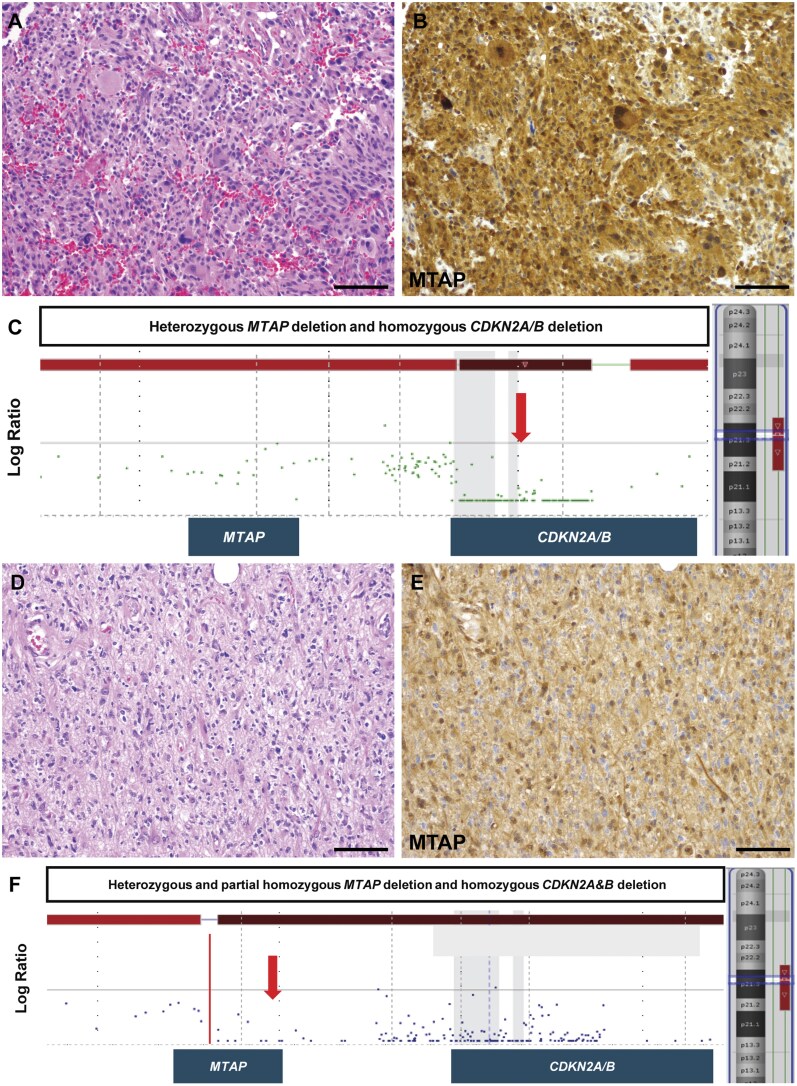
Representative examples of “discordant” MTAP IHC and *CDKN2A/B* status. (A-C) A recurrent oligodendroglioma, IDH-mutant and 1p/19q-codeleted, composed of tumor cells with abundant cytoplasm and scatted multinucleated giant cells (A) with retained MTAP IHC expression (B). OncoScan demonstrates heterozygous deletion of *MTAP* and *CDKN2A&B* homozygous deletion (C). (D-F) Astrocytoma, IDH-mutant, with *CDKN2A&B* homozygous deletion (D) with retained MTAP IHC expression (E). OncoScan shows *MTAP* with heterozygous deletion (proximal) and partial homozygous deletion (distal; denoted by red line) (F). Scale bar, 100 μm.

**Figure 5.**
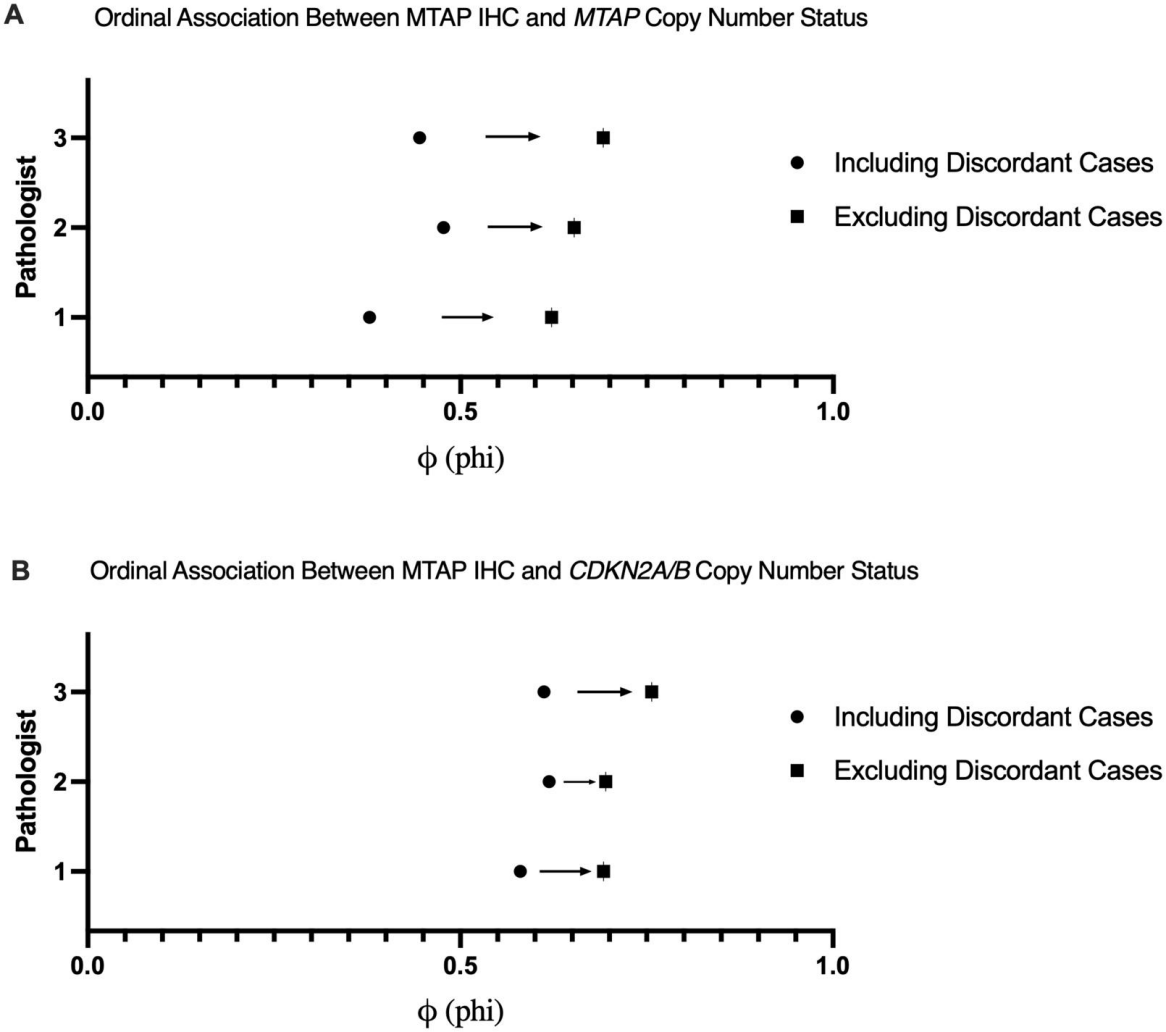
Ordinal/binary association between MTAP IHC and (A) *MTAP* copy number status and (B) *CDKN2A/B* copy number status. Circles show φ (phi) including discordant cases and squares show φ after excluding discordant cases. For each pathologist, the φ association between MTAP IHC with both *MTAP* (A) and *CDKN2A/*B (B) improved with exclusion of the discordant case (arrows indicate change). Loss was defined strictly as IHC stain = 0 and gene copy number = 0 (homozygous deletion). All changes were statistically significant, except for the association change between Pathologist 2 and MTAP IHC:*CDKN2A/B*; p < 0.05 determined by two-tailed permutation test.

Lastly, we sought to determine how tumors with discordant alterations of MTAP and *CDKN2A/B* would affect the correlation between MTAP IHC to both *MTAP* and *CDKN2A/B* copy number status. MTAP protein expression was assessed by IHC in 63 adult-type diffuse gliomas including glioblastoma, IDH-wildtype (n = 15), IDH-mutant astrocytomas (n = 40), and oligodendrogliomas (n = 8). *CDKN2A/B* homozygous deletion was present in 25 tumors (glioblastoma, n = 12; IDH-mutant astrocytomas, n = 11; oligodendroglioma, n = 2). The remaining cases had either *CDKN2A/B* heterozygous deletion (n = 21) or intact *CDKN2A/B* (n = 17). Of the tumors with *CDKN2A/B* alterations, we selectively enriched the cohort with tumors harboring discordant *MTAP* and *CDKN2A/B* deletions (n = 14). Illustrative examples of tumors with concordant *MTAP* and *CDKN2A/B* deletions are shown in Fig. 3, and discordant tumors are shown in Fig. 4. A subset of discordant cases with *CDKN2A/B* homozygous deletion and complete or partial heterozygous *MTAP* deletion showed retained MTAP expression, raising the possibility that the truncated MTAP transcripts may result in retained some degree of protein expression. Therefore, we determined the binary/ordinal association between MTAP IHC expression and *MTAP* copy number status in both the presence and absence of discordant tumors. These findings demonstrate that for three different pathologists the correlation between MTAP IHC and *MTAP* copy number status significantly improved with the exclusion of discordant tumors (Fig. 5A). Further, in the context of MTAP’s utility as surrogate marker of *CDKN2A/B* status, removal of the discordant tumors also improved the correlation between MTAP IHC and *CDKN2A/B* copy number status (Fig. 5B).

## DISCUSSION

This study evaluates the genomic architecture of chromosomal alterations resulting in *CDKN2A/B* and/or *MTAP* deletion in 333 primary adult-type diffuse CNS gliomas. A major finding is the strong correlation at the genomic level between *MTAP* and *CDKN2A/B* deletion. The *CDKN2A/B* heterozygous deleted tumors always co-occurred with *MTAP* heterozygous deletion; tumors with a *CDKN2A/B* homozygous deletion showed concurrent homozygous and/ or heterozygous *MTAP* deletion with only a single tumor lacking concurrent *MTAP* deletion. We also found that tumors with *CDKN2A/B* heterozygous or homozygous deletions often harbor large heterozygous deletions of 9p, which correlated with the high degree of concordance between heterozygous *MTAP* co-deletion. Conversely, the deletions resulting in homozygous *CDKN2A/B* are significantly smaller, which likely explains why a subset of tumors lack concordant homozygous deletion of *MTAP*. Small homozygous deletions of *CDNK2A/B* may also result in tumors harboring a *MTAP* gene with only a partial homozygous deletion. Taken together, these results suggest the initial loss of *CDKN2A/B* and *MTAP* occurs first through larger 9p heterozygous deletions that include both genes and are likely followed by focal “second” hit(s) to the 9p.21-22 region. These results may also indicate why *MTAP* is frequently but not always co-deleted as the “second” hit has propensity to include complete/partial homozygous deletion of *MTAP* (73%). These findings are the first to rigorously evaluate at the genomic level the association between *MTAP* and *CDKN2A/B* loss in adult-type diffuse gliomas and were observed across each of the entities.

As illustrated in Fig. 1, homozygous *CDKN2A/B* deletions may arise as a “second hit” on the background of hemizygous loss, fitting a stepwise model of tumor progression. Therefore, it is conceivable that tumors with *CDKN2A/B* hemizygous deletion would be at higher overall risk of progression relative to copy-neutral tumors. Recent reports suggest that *CDKN2A/B* hemizygous deletion in IDH-mutant diffuse gliomas is associated with a significantly worse overall survival (24, 30, 31). However, the prognostic impact of *CDKN2A/B* hemizygous deletion is not uniform across studies. Ippen et al found no significant difference in overall or progression‑free survival (32). While the prognostic significance of hemizygous *CDKN2A/B* deletions in IDH-mutant gliomas requires additional study, our findings suggest that in diffuse gliomas there is a recurrent pattern chromosome 9p alteration that may prime tumors for a “second hit” resulting in *CDKN2A/B* homozygous deletion.

Among glioblastomas (GBM), Hansen et al reported homozygous deletion of *MTAP* being significantly associated with reduced disease-free survival and that this association was independent of *CDNK2A* alteration status (33). However, Menezes et al did not observe a significant association between *MTAP* deletion and disease-free survival in their cohort (34). In GBM cell-lines, homozygous deletion of *MTAP* and its consequent loss of function is as a molecular alteration that promotes tumorigenesis via the GBM immuno-microenvironment (35). It is notable that in our cohort of 333 tumors, we did not observe *MTAP* deletion in the absence of *CDNK2A/B* co-deletion, while a number of tumors with *CDNK2A/B* homozygous deletions did not harbor concurrent *MTAP* homozygous deletion. It remains to be determined whether the strong concordance between homozygous deletions of *CDKN2A/B* and *MTAP* confers a selective advantage to the tumor cells or may be secondary to a change in overall genomic architecture that predisposes to such a co-deletion.

Lastly, we paired the genomic analysis with MTAP IHC and assessed its association with *MTAP* and *CDKN2A/B* homozygous deletion status. Herein we do not report the sensitivity and specificity for MTAP as a surrogate marker of *CDKN2A/B* status given the selection bias used in our cohort. Other reports provide a direct assessment of the overall sensitivity and specificity in a general practice (21–25). Important technical differences exist between these reports and our approach, including that we utilized a polyclonal MTAP antibody and a distinct IHC thresholding. Our results show that tumors with discordant *MTAP* and *CDKN2A/B* copy number status negatively affect the correlation between MTAP IHC and copy number status of *MTAP* and *CDKN2A/B*. It could be reasonably inferred that MTAP immunostain may show “false negative” results secondary to cases with homozygous *CDKN2A/B* deletion not accompanied by concordant *MTAP* homozygous deletion. Furthermore, the observation that 26% of tumors with homozygous *CDKN2A/B* deletion had only heterozygous *MTAP* loss underscores the need for caution when inferring intact *CDKN2A/B* status based solely on MTAP IHC. Overall, we find that the use and interpretation of MTAP for assessment of *CDNK2A/B* should be carefully considered. Homozygous deletion of *CDKN2A/B* and *MTAP* appear to frequently co-occur and combined use of MTAP and p16 IHC may have clinical utility as an initial screening tool to guide or while detailed molecular testing is being performed. Further investigation of the biological significance of loss of any of these three genes may provide further insight into gliomagenesis and progression.
